# Effect of sutureless securement on hemodialysis catheter-related bloodstream infection

**DOI:** 10.1038/s41598-021-01372-6

**Published:** 2021-11-05

**Authors:** Keiji Fujimoto, Emi Iida, Syo Kumano, Ai Fujii, Hiroki Adachi, Kengo Furuichi, Hitoshi Yokoyama

**Affiliations:** 1 Department of Nephrology, Kanazawa Medical University School of Medicine, 1-1 Daigaku, Uchinada, Ishikawa 920-0293 Japan; 2 Blood Purification Center, Kanazawa Medical University Hospital, Uchinada, Ishikawa Japan; 3 Nursing Services Division, Kanazawa Medical University Hospital, Uchinada, Ishikawa, Japan

**Keywords:** Medical research, Nephrology, Renal replacement therapy

## Abstract

The use of sutureless securement devices during catheterization might reduce the risk of catheter-related bloodstream infection (CRBSI) by suppressing catheter-exit infection and catheter dislodgement. However, the effectiveness of these devices in reducing CRBSI risk when securing hemodialysis catheters has not been explored. This single-center retrospective observational study examined 211 non-tunneled hemodialysis catheters (NTHCs) from 110 hemodialysis inpatients, of which 121 were secured using conventional skin sutures (Suture group) and 90 with GRIP-LOK (GRIP-LOK group). The stabilized inverse probability of treatment (SIPT)-weighting method was used to generate a new population (SIPT-weighted model) without group differences for each of the 12 predictors of CRBSI development (i.e., age, sex, dialysis history, concomitant acute kidney injury or diabetes, concurrent use of immunosuppressant drugs or aspirin, NTHC insertion site, methicillin-resistant Staphylococcus aureus, carriage, bacteremia event within 3 months before catheterization, hemoglobin level, and serum albumin titer). The effect of GRIP-LOK compared with sutures on CRBSI in the SIPT-weighted model was evaluated using univariate SIPT-weighted Cox proportional regression analysis, which showed a significant CRBSI suppression effect of GRIP-LOK compared with sutures (hazard ratio: 0.17 [95% CI 0.04–0.78], p = 0.023).　GRIP-LOK affords a lower risk of CRBSI due to indwelling NTHCs than conventional securement using sutures.

## Introduction

Infections result in the death of dialysis patients at rates nearly 50- to 80-fold higher than the rates in the general population^1, 2^, thus making this an urgent issue that must be addressed by dialysis care providers. Catheter-related bloodstream infection (CRBSI) is a classic complication of intravenous catheter placement and is responsible for most infection-related deaths in hemodialysis patients^3^. Prevention is the first priority, given the high fatality rate of CRBSI, which results in the death of approximately between 4 and 25% of patients in whom it occurs^4^.

The US Centers for Disease Control and Prevention (CDC) suggested using a sutureless securement device to affix catheters to the skin to reduce CRBSI risk^5^. However, the only study presented to support this proposal was a single randomized controlled trial of peripherally inserted central venous catheters^6^; no such evidence has been established for hemodialysis catheters.

Sutureless securement devices can be classified into two main types: subcutaneous and adhesive. GRIP-LOK™ (TIDI Products, Neenah, WI, USA) belongs to the latter category: the device secures catheters using a locking mechanism and is attached to the skin by an adhesive pad^7^. Skin punctures from suturing increase CRBSI risk because they breach the protective nature of the skin barrier^5^. In addition, micro-movements of the catheter at the exit site pose a significant risk of infection, which can be exacerbated by poor suturing technique at the catheter hub^7^. The GRIP-LOK uses hypoallergenic medical adhesive, which reduces the risk of skin irritation and shows strong adhesion to the skin. From the above, it is speculated that GRIP-LOK is more effective in preventing CRBSI than suturing.

In this retrospective cohort study, we examined whether using a GRIP-LOK device was more effective than skin sutures at preventing CRBSI in patients undergoing hemodialysis via non-tunneled hemodialysis catheters (NTHCs). This observation is expected to be a starting point to encourage further research to verify this effect, such as with randomized control studies, with the ultimate goal of promoting the use in clinical practice for the benefit of dialysis patients.

## Results

### Clinical background

**Figure 1:**
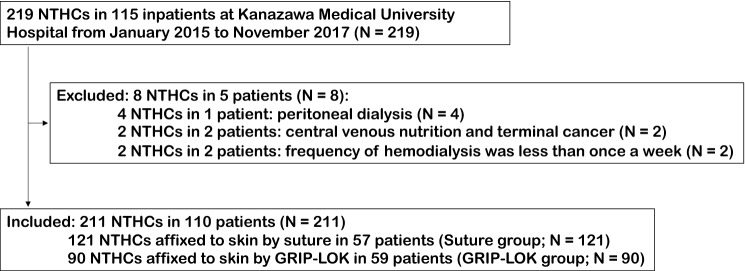
Flowchart of the study patient selection process. In this study, there were patients who experienced multiple catheter insertion events. The sum of the number of patients in the suture group and the number of patients in the non-suture group was greater than the total number of patients because some patients experienced both suturing and non-suturing for each catheter insertion event. Therefore, *n* in this study is not the number of patients, but the number of catheters. NTHC, non-tunneled hemodialysis catheter.

Among 219 NTHCs (115 inpatients) in the initial study, eight NTHCs (5 inpatients) were removed as they met the exclusion criteria, leaving 211 NTHCs (110 inpatients) in the final analysis dataset (Fig. 1). Catheters were secured to the skin using sutures in 121 NTHCs (57 inpatients) and a GRIP-LOK device in 90 NTHCs (59 inpatients).

Table 1 shows patients' background characteristics at each catheter insertion event in the crude population and the SIPT-weighted model. The 12 clinical parameters listed in Table 1 are predictors of the development of CRBSI and are covariates (confounders or potential confounders) that should be adjusted for when evaluating the efficacy of GRIP-LOK compared to sutures in reducing the development of CRBSI. Most covariates were not balanced between the Suture and GRIP-LOK groups in the crude population (std. diff. ≥ 0.10).

**Table 1:** Patients’ background characteristics at each catheter insertion event in the crude population and the SIPT-weighted model.

	Crude population				SIPT-weighted model		
	All (n = 211)	Suture group (n = 121)	GRIP-LOK group (n = 90)	Standardized difference	Suture group (n = 124)	GRIP-LOK group (n = 87)	Standardized difference
Age, mean (SD), years	70.5 (14.0)	69.5 (14.6)	71.8 (13.0)	0.17	70.6 (14.1)	69.7 (15.2)	0.06
**Sex, no. (%)**							
Males	99 (46.9)	55 (45.5)	44 (48.9)	0.07	60 (48.5)	39 (44.5)	0.08
Females	112 (53.1)	66 (54.5)	46 (51.1)		64 (51.5)	48 (45.5)	
Dialysis vintage, mean (SD), months	38.6 (92.6)	38.1 (91.0)	39.2 (95.2)	0.01	35.5 (85.8)	32.7 (82.7)	0.03
Acute kidney injury, no. (%)	21 (10.0)	10 (8.3)	11 (12.2)	0.13	14 (11.3)	11 (12.2)	0.03
Diabetes, no. (%)	95 (45.0)	60 (49.6)	35 (38.9)	0.22	55 (44.5)	37 (42.4)	0.04
Immunosuppressants use, no. (%)	48 (22.7)	28 (23.1)	20 (22.2)	0.02	26 (21.3)	19 (21.3)	0.001
**Catheter insertion site, no. (%)**							
Femoral vein	45 (21.3)	29 (24.0)	16 (17.8)	0.15	27 (21.7)	17 (19.8)	0.05
Internal jugular vein	166 (78.7)	92 (76.0)	74 (82.2)		97 (78.3)	70 (80.2)	
MRSA carriage, no. (%)	69 (32.7)	36 (29.8)	33 (36.7)	0.15	39 (31.7)	26 (30.0)	0.04
Bacteremia in the 3-month period before catheter insertion, no. (%)	134 (63.5)	88 (72.7)	46 (51.1)	0.46	74 (59.6)	52 (59.3)	0.01
Aspirin use, no. (%)	55 (26.1)	41 (33.9)	14 (15.6)	0.44	31 (25.1)	19 (21.2)	0.09
Hb, mean (SD), g/dL	9.7 (1.9)	9.5 (1.6)	10.0 (2.1)	0.24	9.6 (1.6)	9.8 (2.1)	0.09
Serum albumin, mean (SD), g/dL	2.9 (0.7)	2.8 (0.7)	2.9 (0.7)	0.16	2.9 (0.7)	2.9 (0.7)	0.02

The 12 clinical parameters listed in Table 1 are predictors of the development of catheter-related bloodstream infection (CRBSI) and are covariates (confounders or potential confounders) that should be adjusted for when evaluating the efficacy of GRIP-LOK compared to sutures in reducing the development of CRBSI. In the newly generated population from the crude population by the SIPT-weighting method (SIPT-weighted model), group differences for all covariates were eliminated (Standardized difference < 0.10 for all).

*SIPT* stabilized inverse probability of treatment, *MRSA* methicillin-resistant *Staphylococcus aureus.*

Patients in the Suture group had disproportionately higher rates of diabetes, femoral vein placement, and bacteremia in the 3 months before catheterization, as well as lower average Hb and serum albumin levels. Patients in the GRIP-LOK group tended to be older and had disproportionately higher rates of acute kidney injury (AKI) and methicillin-resistant *Staphylococcus aureus* (MRSA) carriage. In addition, aspirin—a factor reported to reduce CRBSI risk^8^—was more likely to be taken by members of the Suture group.

The SIPT-weighting method was used to generate a new population (SIPT-weighted model), without group differences for each of the 12 predictors of CRBSI development, as shown Table 1. In the SIPT-weighted model using propensity scores (PSs) (c-statistic = 0.74 [95% confidence interval [CI] 0.67‒0.81]), all covariates were successfully balanced (std. diff < 0.10 for all). The kernel density estimates of the PS distributions of the two treatment groups before and after SIPT-weighting are shown in Supplementary Fig. S2 online. The PS distributions of the two groups clearly showed greater overlap after SIPT-weighting.

### GRIP-LOK use is associated with reduced CRBSI risk

**Figure 2:**
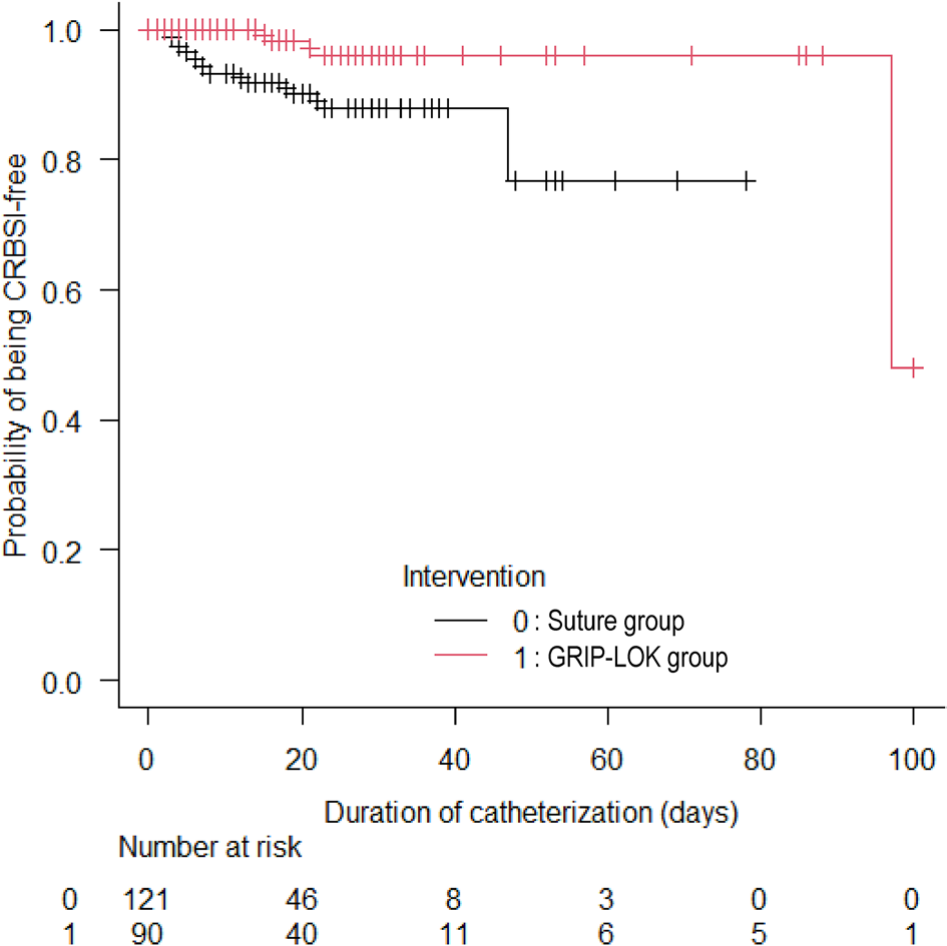
Kaplan‒Meier plot of the probability of avoidance of catheter-related bloodstream infection (CRBSI) events in the crude population. In total, 14 CRBSI events occurred within 100 days after non-tunneled hemodialysis catheter insertion: 11 in the Suture group and three in the GRIP-LOK group.

CRBSI incidences (events/1000 catheter days) in the crude population were 5.25 in the Suture group and 1.44 in the GRIP-LOK group: the median time to onset after catheterization was 9.5 days (interquartile range [IQR]: 4.0–20.0). Figure 2 shows the cumulative incidences of CRBSI in the two groups for the crude population. In total, 14 CRBSI events (Suture group: 11 events, GRIP-LOK group: 3 events) were recorded: six events (Suture group: 5 events, GRIP-LOK group: 1 events) were caused by methicillin-resistant coagulase-negative staphylococci, three events in the Suture group by methicillin-sensitive *Staphylococcus aureus*, and one event each due to MRSA (Suture group), *S. epidermidis* (GRIP-LOK group), *Propionibacterium acnes* (Suture group), *Escherichia coli* (Suture group), and *Enterococcus faecalis* (GRIP-LOK group).

The SIPT-weighted model is a population in which the values or distributions of confounding factors are adjusted to be the same between the two groups, as shown in Table 1. Therefore, confounding adjustment is no longer necessary to compare the effectiveness in preventing the development of CRBSI between the two groups in the SIPT-weighted model, and the effectiveness was examined by univariate SIPT-weighted Cox proportional hazards regression analysis. In the SIPT-weighted model, GRIP-LOK was associated with a significant reduction in CRBSI incidence compared with sutures (Hazard ratio [HR] in the SIPT-weighted model: 0.17 [95% CI 0.04–0.78], p = 0.023) (Main analysis).

Next, the results of main analysis were validated in a multivariate Cox proportional-hazards regression model for the crude population, using PS and the catheter securement method as explanatory variables. GRIP-LOK was again associated with a significantly lower CRBSI incidence than sutures, corroborating the treatment effect (PS-adjusted HR 0.20 [95% CI 0.04–0.95], p = 0.043), as shown in Table 2.

**Table 2:** A multivariate Cox proportional-hazards regression model for predictive of time to CRBSI onset in the crude population.

Explanatory variables	Hazard ratio [95% confidence interval]	*P* value
Catheter securement method (Suture: 0, GRIP-LOK: 1)	0.20 [0.04–0.95]*	0.043
Propensity scores	1.28 [0.08–19.7]	0.858

Propensity scores was calculated from age, sex, dialysis history, AKI, diabetes, immunosuppressive drugs, catheterization site, MRSA carriage, bacteremic event within 3 months before catheterization, and aspirin.

*CRBSI* catheter-related bloodstream infection.

*Propensity scores—adjusted Hazard ratio.

**Figure 3:**
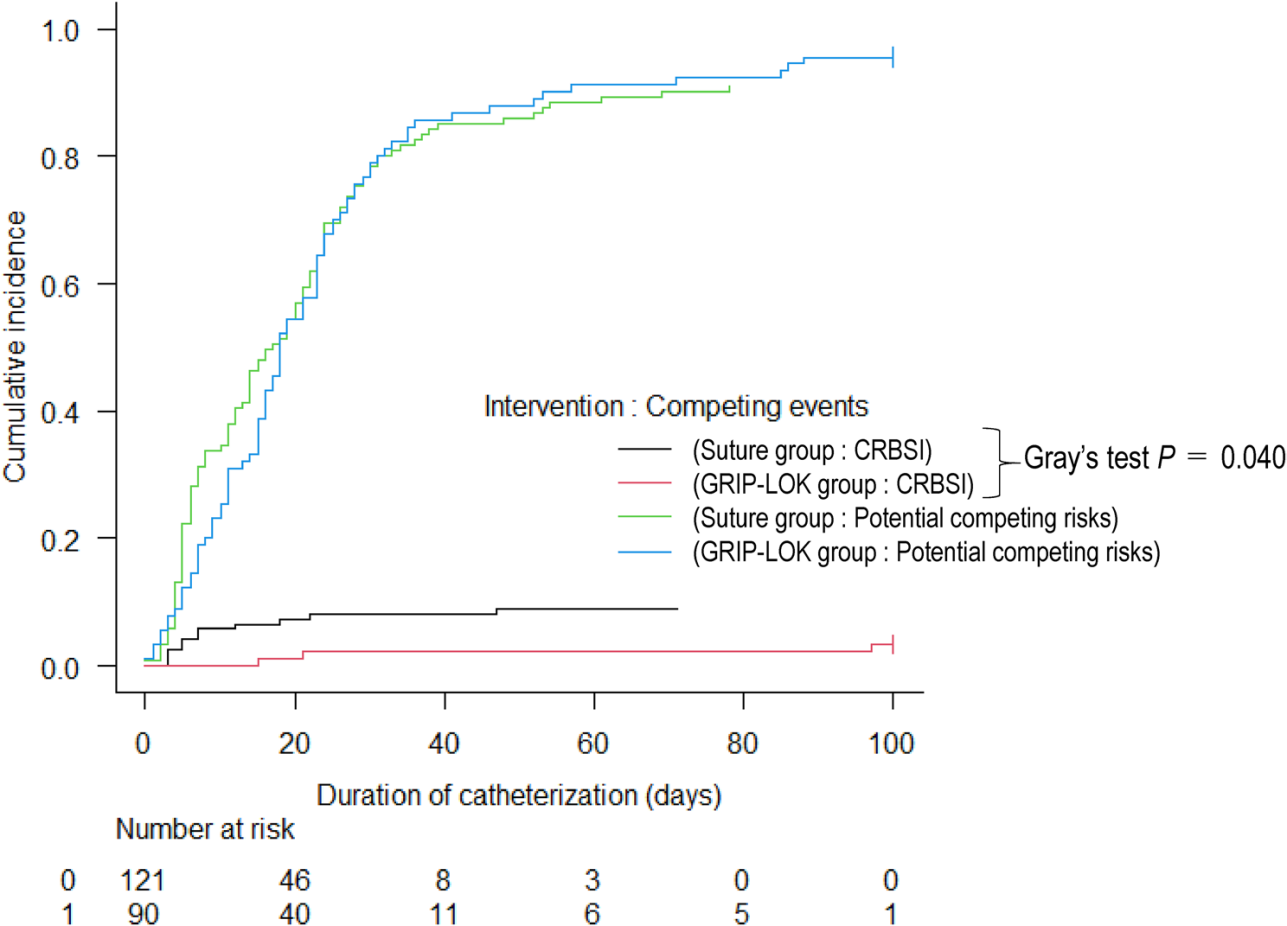
Cumulative incidence curve of catheter-related bloodstream infection (CRBSI) events and potential competitive risks in the crude population. The potential competing risks are (i) catheter removal in acute kidney injury due to recovery of kidney function and no longer need for dialysis; (ii) catheter removal in end-stage kidney disease due to resolution of the arteriovenous (AV) fistula problem, allowing dialysis with an AV fistula; and (iii) unexpected catheter dislodgement.

There were four reasons for "censoring observations" in this study: (i) in acute kidney injury (AKI), the catheter was removed because kidney function was restored and dialysis was no longer needed; (ii) in end-stage kidney disease (ESKD), the catheter was removed because the arteriovenous fistula (AV fistula)problem was resolved and dialysis with an AV fistula was possible; and (iii) there was unexpected catheter dislodgment [171 NTHCs for (i) and (ii), 25 NTHCs for (iii)]. Because there was concern that these censorings might be potential competing risks in this study, cumulative incidence function of CRBSI, which allows for the estimation of the impact of CRBSI when multiple competing events, was investigated. In the crude population, the cumulative incidence function of CRBSI in GLIP-LOK group was significantly lower than that in the suture group (Gray’s test: p = 0.040), as shown in Fig. 3. In addition, Fine-Gray subdistribution hazard analysis, one of the competing risk regression analyses conducted on the SIPT-weighted model (i.e. SIPT-weighted Fine-Gray subdistribution hazard analysis), showed that the GRIP-LOK group significantly reduced the incidence of CRBSI compared to the suture group (subdistribution HR 0.16 [95%CI 0.04–0.66], p = 0.012).

No patients were lost to follow-up during the catheterization period. In addition, no patients were lost to follow-up due to death. No glue allergy was detected in the GRIP-LOK group, and no patients died during the catheterization period.

### GRIP-LOK use is associated with a reduced risk of catheter exit site infection

**Figure 4:**
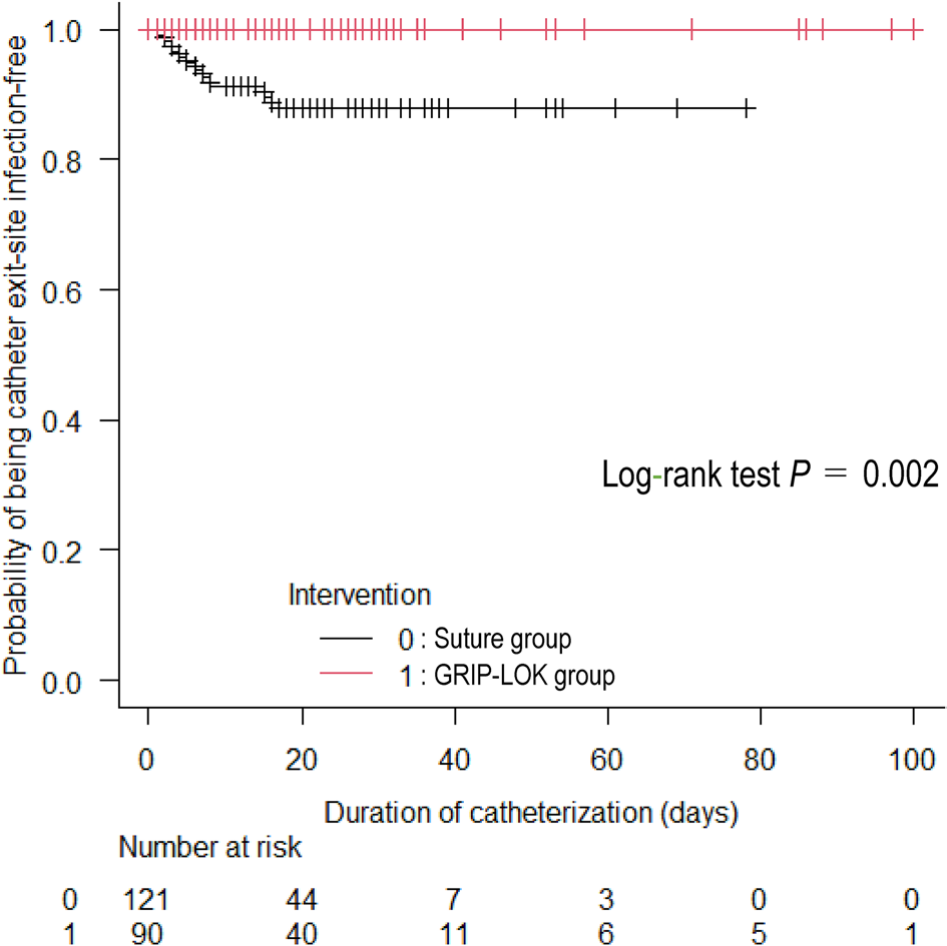
Kaplan‒Meier plot of the probability of avoidance of hemodialysis catheter exit site infection events in the crude population. The cumulative percentage of catheter exit site infection was significantly lower in the GRIP-LOK group than in the Suture group.

Catheter exit site infection incidences (events/1000 catheter days) in the crude population were 5.43 in the Suture group and 0 in the GRIP-LOK group: the median time to onset after catheterization was 5.0 days (IQR 3.0–7.8). The GRIP-LOK group had a significantly lower cumulative incidence of exit site infection than the Suture group (log-rank test: p = 0.002, Fig. 4). In total, 11 events were recorded; eight of these cases later developed CRBSI (72.7%), after a median interval of three days (IQR 1.0–5.0). In addition, eight of the 14 recorded events of CRBSI were preceded by exit site infection (57.1%).

### GRIP-LOK use is associated with a reduced risk of catheter dislodgment due to loosening from the skin

Catheter dislodgment incidences (events/1000 catheter days) in the crude population were 10.98 in the Suture group and 0.96 in the GRIP-LOK group. GRIP-LOK use was associated with a significant reduction in the catheter dislodgment risk in Cox proportional-hazards regression models of the crude population (uncorrected, HR 0.09 [95% CI 0.02–0.37], p = 0.001; adjusted for age and insertion site, HR 0.10 [95% CI 0.02–0.43], p = 0.002) (see Supplementary Table S1 online).

## Discussion

This study demonstrated that the use of a sutureless securement device is associated with reduced CRBSI risk from hemodialysis catheters; this has not been reported previously.

CRBSI primarily occurs with short-term indwelling catheters when the resident skin flora invades the skin via the percutaneous insertion site^9–11^. Sutureless securement devices likely reduce bacterial colonization because they do not compromise the skin around the insertion site, unlike sutures^6^. Species common in skin flora were among the primary pathogenic bacteria observed in our study population: 72.7% of catheter exit site infection events later developed into CRBSI. It is noteworthy that not a single case of obvious exit site infection occurred in the GRIP-LOK group, which is likely to have led to the lower incidence of CRBSI in these patients. One potential reason for these prophylactic effects is the ease with which GRIP-LOK devices can be removed, allowing staff to disinfect the entire catheterization site, including underneath the catheter hub, during routine cleaning and care of the insertion site. When a catheter is secured by sutures, the skin underneath the hub is difficult to access, making it prone to inadequate disinfection^7^.

Catheter dislodgment due to loosening from the skin was also found to occur significantly less frequently in the GRIP-LOK group compared with that in the Suture group. This appears to indicate that GRIP-LOK has a superior ability to secure catheters compared with conventional skin sutures. Indeed, some reports have noted that such adhesive securement devices—which tightly adhere to the skin without the use of sutures—can more effectively minimize catheter movement due to their high stability^12^. Since even slight disturbances of a catheter at the exit site, due to inadequate securement, can increase the catheter infection risk^13^, we surmise that the GRIP-LOK device prevents CRBSI from occurring by keeping such movements to a minimum.

Our study has two major strengths. The first was our confirmation of associations between GRIP-LOK usage and reduced CRBSI risk by using four different statistical analyses. The finding that the four approaches yielded nearly identical results substantiates the robustness of our findings. Among the statistical methods used in this study, SIPT-weighted competing risk analysis may provide a more accurate estimate of the cumulative incidence of CRBSI because it considers potential competing risks in addition to measured confounders. The results of the competing risk analysis and the results of the main analysis that did not take competitive risk into account were the same, which suggests that the presence of potential competitive risks did not bias the results of the main analysis. The second was the high internal validity maintained in our main analysis: catheters were placed at a single center, according to a consistent care and management protocol, and all major predictors for CRBSI reported in the literature were addressed by our model as potential confounders. These included age^14, 15^, sex^14, 16^ dialysis history^17^, AKI^18^, diabetes^19^, immunosuppressive drugs^20^, catheterization site^21–23^, MRSA carriage^24^, bacteremic event within 3 months before catheterization^24^, aspirin^8^, Hb^20, 25^, and serum albumin titer^14–16, 20, 26^.

Conversely, our study has three major limitations. First, we only examined a small number of cases at a single institution, which limits the generalizability of our findings. Second, due to its retrospective observational design, our main analysis could not control for unknown confounding variables. Third, the choice of whether to use sutures or GRIP-LOK was at the discretion of the treating physician. We speculate that there was a tendency for older treating physicians to prefer the traditional skin sutures they had been accustomed to for many years, but it is unclear what bias this may have had on outcome assessment. Accordingly, our findings need to be further validated in a future joint, multicenter, randomized controlled trial with a larger sample size.

In conclusion, our findings show that GRIP-LOK can reduce the risk of CRBSI in hemodialysis patients inserted with NTHCs compared with conventional securement using sutures.

## Methods

### Study participants

The initial study dataset comprised the medical records of 219 catheterization events (115 inpatients) for hemodialysis using an NTHC between January 2015 and November 2017 at the Kanazawa Medical University Hospital Department of Nephrology, as part of treatment for end-stage kidney disease or AKI. As a rule of our hospital, patients undergoing NTHCs were managed in the hospital, and were not outpatients. Thus only inpatients were eligible for this study. Candidate patients were excluded if they were under 18 years of age at the time of catheterization; undergoing hemodialysis less frequently than once per week; undergoing concurrent peritoneal dialysis; receiving central parenteral nutrition; or had terminal cancer.

Clinical data for the following variables were obtained from patients' electronic medical records: NTHC securement method (sutures vs GRIP-LOK); occurrence of CRBSI, catheter exit site infection, or catheter dislodgment due to loosening from the skin (Y/N); age, sex, and dialysis history; concomitant AKI or diabetes (yes/no), concurrent use of immunosuppressive drugs or aspirin; NTHC insertion site (internal jugular vein/femoral vein); MRSA carrier status; bacteremia in the 3 months before NTHC insertion; hemoglobin level (Hb); and serum albumin titer.

The study population did not include incarcerated volunteers or any individuals with cognitive developmental delay. All patients provided written informed consent, and the study was conducted according to the principles of the Declaration of Helsinki. The study protocol was reviewed and approved by the Kanazawa Medical University Hospital Ethics Committee (ethics review no. 103).

### Study design and endpoints

The study was a single-center, retrospective cohort study. The primary outcome was the incidence of CRBSI within 100 days of NTHC insertion. Secondary outcomes were the incidences of catheter exit site infection and dislodgment due to loosening from the skin, both within 100 days of NTHC insertion.

We diagnosed CRBSI and catheter exit site infection per CDC guidelines^27^. CRBSI was defined as bacteremia associated with NTHC, with all of the following elements: 1. In the case of common commensals, such as coagulase-negative *Staphylococcus*, both catheter and peripheral blood cultures growing the same organism; in the case of all other organisms, at least one positive blood culture (catheter hub or peripheral blood or both); 2. Clinical manifestations of infection (one or more of the following: fever > 38℃, chills, or hypotension); 3. No other apparent source of the bloodstream infection; 4. Catheter in use within 48 h of the CRBSI.

Catheter exit site infection was defined as erythema, induration, and/or tenderness within 2 cm of the catheter exit site. It could be associated with other signs and symptoms of infection, such as fever or purulent drainage emerging from the exit site, with or without concomitant bloodstream infection. Catheter dislodgment was defined as accidental removal or movement that resulted in the loss of function and the need for insertion of a new catheter.

### Dialysis catheter care and management protocol

The NTHCs used were polyurethane urokinase-coated double lumen catheters (NIPRO, Osaka, Japan): 12 Fr × 15 cm for internal jugular vein insertion or 12 Fr × 25 cm for femoral vein insertion. The skin at the intended insertion site was disinfected with 1% chlorhexidine alcohol solution (CH-AL), after which the catheter was inserted under ultrasonographic guidance while following maximal sterile barrier precautions. Once inserted, the catheter was secured by either sutures or a GRIP-LOK device.

The GRIP-LOK catheter-locking mechanism consists of two components—an adhesive flap and a Magic Tape-like hook-and-loop fastener—between which the catheter hub is immobilized (see Supplementary Fig. S1 online). Once the catheter was secured, the insertion site was covered using a transparent film dressing. Once a week, the site was disinfected with 1% CH-AL, and the GRIP-LOK device and dressing were replaced.

Patients in whom suturing was performed were checked for looseness or deterioration in the stitches: if necessary, the catheter was re-secured using a new suture thread.

Once hemodialysis was complete, the catheter was flushed with heparin sodium, and the connector(s) was disinfected with cotton soaked in 80% ethanol and then fitted with an injection cap.

Patients were provided with standardized education regarding prevention topics including vascular access care, hand hygiene, risks related to catheter use, recognition of signs of infection, and instructions for access management when they are away from their dialysis unit. In addition, the nurse in charge of the patient in the ward observed and maintained the hygiene of the catheter insertion site at least once a day.

### Statistical analysis

Numerous factors that may affect CRBSI risk have been reported in previous research: these include age^14, 15^, sex^14, 16^ dialysis history^17^, AKI^18^, diabetes^19^, immunosuppressive drugs^20^, catheterization site^21–23^, MRSA carriage^24^, bacteremic event within 3 months before catheterization^24^, aspirin^8^, Hb^20, 25^, and serum albumin titer^14–16, 20, 26^. Since patient treatment groups were not randomized, our models needed to control for these covariates. First, PSs for each patient and treatment (i.e., catheter securement method; suture: 0, GRIP-LOK: 1) were calculated using a multiple logistic regression model incorporating the covariates above as explanatory variables. The power of this PS model to discriminate between treatments was evaluated in terms of the c-statistic.

Next, covariates were balanced between the two groups based on the SIPT-weighting method using PS^28^. The covariate balance between the groups in the SIPT-weighted model was evaluated in terms of standardized difference (std. diff.), with values < 0.1 considered to indicate a good balance. In the SIPT-weighted model, the average treatment effect of the GRIP-LOK compared to suture securement—i.e., HR in the SIPT-weighted model—was evaluated using univariate SIPT-weighted Cox proportional-hazards regression, with catheter securement method as explanatory variables (Main analysis).

Next,the following three sensitivity analyses validated the results of the main analysis.

As the first sensitivity analysis, to confirm that the results of our main analysis were not dependent on the processing method (SIPT-weighting), they were corroborated in the crude (unmatched) population using multivariate Cox proportional-hazards regression, with PS and catheter securement method as explanatory variables. The average treatment effect of GRIP-LOK relative to suture securement was evaluated in terms of a PS-adjusted HR.

A second and a third sensitivity analyses were conducted to consider the potential competing risks. Gray's test^29^ is used to evaluate hypotheses of equality of CRBSI-specific cumulative incidence functions between suture group and GRIP-LOK group. In addition, the SIPT-weighted Fine-Gray subdistribution hazard analysis^29, 30^, which takes into account potential competing risks in addition to measured confounders, was performed.

All *p* values were two-sided and *p* values of < 0.05 were considered statistically significant. All *p* values reported in this work are nominal *p* values. All statistical analyses were performed with EZR (Saitama Medical Center, Jichi Medical University, Saitama, Japan)^31^.

## Supplementary Information

Supplementary Information.

## Data Availability

The datasets analyzed during the current study are available from the corresponding author on reasonable request.
