# Supplementary material for: Effect of sutureless securement on hemodialysis catheter-related bloodstream infection
Source: Sci Rep. 2021 Nov 5;11:21771. doi: 10.1038/s41598-021-01372-6 (PMC8571352; doi:10.1038/s41598-021-01372-6)
Supplement: Supplementary file 1 — Supplementary Information. [file 41598_2021_1372_MOESM1_ESM.doc]

**Supplementary Table S1.** Cox regression analyses for catheter dislodgment using crude data

|  | Univariate | | Multivariate | |
| --- | --- | --- | --- | --- |
|  | HR [95% CI] | *p*-value | HR [95% CI] | p-value |
| Catheter fixation methods  (0: Suture, 1: GRIP-LOK) | 0.09  [0.02‒0.37] | 0.001 | 0.10  [0.02‒0.43] | 0.002 |
| Catheter insertion site  (0: Internal jugular vein, 1: Femoral vein) | 7.34  [3.28‒16.43] | <0.001 | 4.77  [2.03‒11.2] | <0.001 |
| Age | 1.06  [1.02‒1.11] | 0.007 | 1.05  [1.00‒1.09] | 0.035 |

Abbreviations: CI: confidence interval; HR: hazard ratio

**Supplementary Figures**


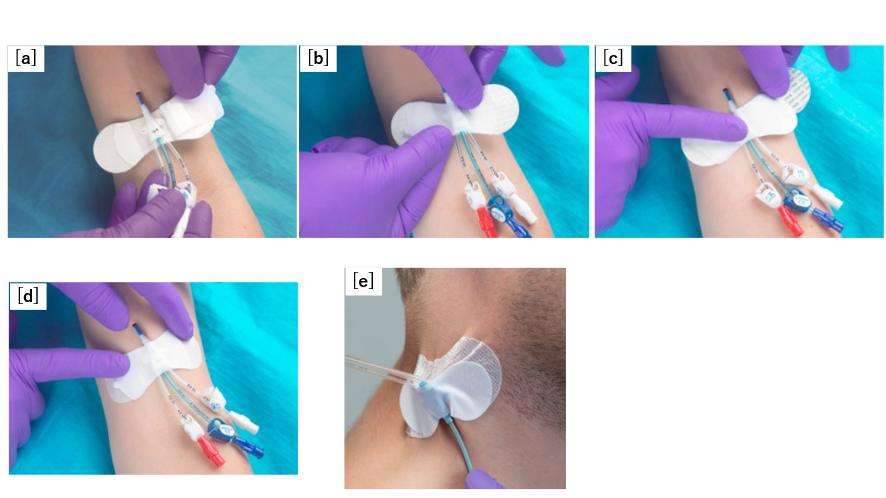


**Supplementary Figure S1. GRIP-LOK securement procedure and behavior under tension**

GRIP-LOK devices were placed according to steps [a] to [d] described below. [a] Open the top flap at one end and peel the liner off its underside. Slide GRIP-LOK under the catheter, positioning the hub at the center of the adhesive side. [b] Close the flap to cover and secure the catheter, pressing down around the catheter and lumen(s) such that it is firmly attached to the adhesive side. [c] Keeping GRIP-LOK and the catheter in place with one hand, and peel off the liner from the underside of one end of the device and affix it to the skin. [d] Next, peel off the liner from the underside of the other end and affix it to the skin to secure the device. [e] GRIP-LOK is made of soft and flexible, latex-free, breathable material, which minimizes catheter slippage due to the horizontal and vertical tension applied to the catheter hub.


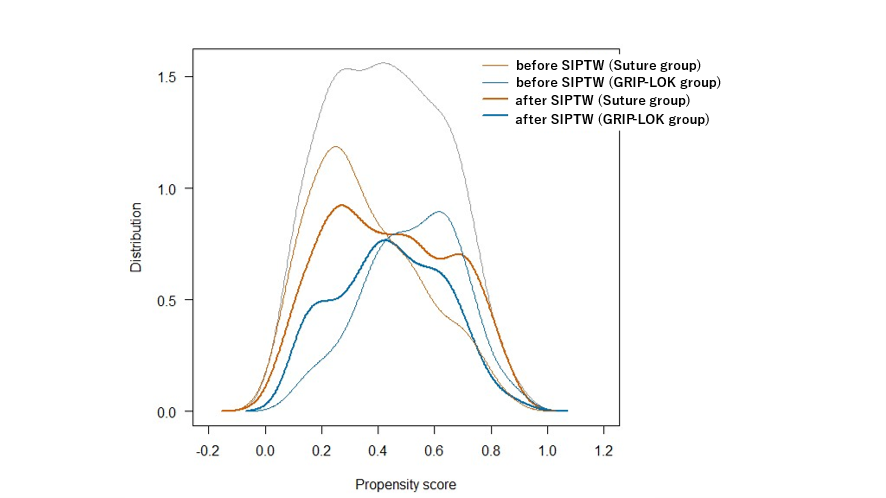


**Supplementary Figure S2. Propensity score distributional overlap before and after stabilized inverse probability treatment-weighting (SIPTW)**

Graphs are kernel density estimate (KDE) plots of propensity score (PS) distributions of the study population and the two treatment groups. The PS distributions of the Suture and GRIP-LOK groups clearly show greater overlap after SIPTW than before SIPTW (in the crude population).
